# Algal Oil Mitigates Sodium Taurocholate-Induced Pancreatitis by Alleviating Calcium Overload, Oxidative Stress, and NF-κB Activation in Pancreatic Acinar Cells

**DOI:** 10.3390/cimb46050267

**Published:** 2024-05-07

**Authors:** Yi Fang, Sung-Yen Lin, Chung-Hwan Chen, Hui-Chen Lo

**Affiliations:** 1 Department of Nutritional Science, Fu Jen Catholic University, New Taipei City 24205, Taiwan; 14k-4uyf7fejcp@dingtalk.com; 2 Department of Orthopaedic Surgery, Kaohsiung Medical University Hospital, Kaohsiung Medical University, Kaohsiung 80708, Taiwan; tony8501031@gmail.com (S.-Y.L.); hwan@kmu.edu.tw (C.-H.C.); 3 Orthopaedic Research Centre, Kaohsiung Medical University, Kaohsiung 80708, Taiwan; 4 Regenerative Medicine and Cell Therapy Research Centre, Kaohsiung Medical University, Kaohsiung 80708, Taiwan; 5 School of Medicine, College of Medicine, Kaohsiung Medical University, Kaohsiung 80708, Taiwan; 6 School of Post-Baccalaureate Medicine, College of Medicine, Kaohsiung Medical University, Kaohsiung 80708, Taiwan; 7 Department of Orthopedics, Kaohsiung Medical University Gangshan Hospital, Kaohsiung 820, Taiwan; 8 Department of Orthopaedics, Kaohsiung Municipal Ta-Tung Hospital, Kaohsiung 80145, Taiwan; 9 Ph.D. Program in Biomedical Engineering, College of Medicine, Kaohsiung Medical University, Kaohsiung 80708, Taiwan; 10 Institute of Medical Science and Technology, National Sun Yat-sen University, Kaohsiung 804, Taiwan; 11 Graduate Institute of Animal Vaccine Technology, College of Veterinary Medicine, National Pingtung University of Science and Technology, Pingtung 912, Taiwan

**Keywords:** acute pancreatitis, algal oil, intracellular calcium concentration, oxidative stress, inflammatory mediators, nuclear factor kappa-B

## Abstract

Acute pancreatitis (AP) is characterized by elevated intracellular Ca^2+^ concentrations, mitochondrial dysfunction, and oxidative stress in pancreatic acinar cells. Algal oil (AO) has demonstrated antioxidant and anti-inflammatory properties. This study aims to explore the effects of algal oil on the microenvironment of AP. Rat pancreatic acinar AR42J cells were pretreated with AO containing 0, 50, 100, or 150 μM of docosahexaenoic acid (DHA) 2 h prior to AP induction using sodium taurocholate (STC). After 1 h of STC treatment, AR42J cells exhibited a significant increase in intracellular Ca^2+^ concentration and the production of amylase, lipase, reactive oxygen species, and pro-inflammatory mediators, including tumor necrosis factor-α and interleukin-6. These STC-induced increases were markedly reduced in cells pretreated with AO. In comparison to cells without AO, those treated with a high dose of AO before STC exposure demonstrated a significant increase in mitochondrial membrane potential and a decrease in lipid peroxidation. Furthermore, STC-activated nuclear factor kappa-B (NF-κB) was attenuated in AO-pretreated cells, as evidenced by a significant decrease in activated NF-κB. In conclusion, AO may prevent damage to pancreatic acinar cells by alleviating intracellular Ca^2+^ overload, mitigating mitochondrial dysfunction, reducing oxidative stress, and attenuating NF-κB-targeted inflammation.

## 1. Introduction

Acute pancreatitis (AP), an inflammatory condition of the pancreas characterized by exaggerated oxidative stress, is closely associated with gallstones and alcohol abuse [1]. It can lead to systemic inflammatory response syndrome and multiple organ dysfunction [2]. Approximately 30% of mortality is linked to the severe form of AP [3]. The mechanisms behind AP development are not fully understood, but studies suggest that a series of cellular changes, such as intracellular Ca^2+^ overload, mitochondrial dysfunction, excessive oxidative stress, and an exacerbated inflammatory response, play a role in the pathogenesis of AP in the pancreatic acinar cells [4,5,6].

In the initial stage of AP, excess bile acid activates abnormal Ca^2+^ release from the endoplasmic reticulum of pancreatic acinar cells, leading to intracellular Ca^2+^ overload in the mitochondria. This overload subsequently results in increased mitochondrial membrane permeability and depolarization [5]. Mitochondrial dysfunction in the pancreas of mice with AP could be induced either by mitochondrial Ca^2+^ overload or through Ca^2+^ overload-independent animal models, highlighting its central role in the pathogenesis of AP [7]. The damaged mitochondria are associated with elevated oxidative stress, as evidenced by the increased production of reactive oxygen species (ROS) and lipid peroxidation in pancreatic acinar cells [8,9]. Subsequently, the nuclear factor-kappa B (NF-κB) signaling pathway is activated to trigger the transcription and production of pro-inflammatory cytokines, such as tumor necrosis factor-α (TNF-α) and interleukin-6 (IL-6). This cascade of events amplifies inflammation and initiates the development of AP [9,10,11]. In AP induced by caerulein and lipopolysaccharide, Lu et al. [12] observed elevated mRNA levels of TNF-α and IL-6, along with activation of the NF-κB p65 signaling pathway in pancreatic acinar cells. These studies indicate that the development of AP involves intracellular Ca^2+^ overload-initiated mitochondrial disorders and the subsequent inflammatory response in the pancreas.

There is currently no available drug for the treatment of AP, and the recommended management is limited to supportive care [13,14]. As an anti-inflammatory agent, aspirin has been noted to decrease TNF-α, IL-6, and nuclear NF-κB p65 levels in the pancreatic acinar cells of AP mice [12]. Recently, fish oils, particularly docosahexaenoic acid (DHA) and eicosapentaenoic acid (EPA), have emerged as potential supplements for alleviating AP due to their antioxidant and anti-inflammatory properties [14].

Although deep-water fish are a primary source of DHA and EPA, concerns about overfishing and potential contamination pose challenges to the sustainability of fish oils. Notably, the major sources of DHA and EPA in marine fish come from algae and phytoplankton, suggesting that cultivated microalgae serve as an easily accessible alternative source of these beneficial fatty acids. Algal oil, a plant-based source of n-3 polyunsaturated fatty acids (PUFAs), offers several advantages over fish oil. It is suitable for vegetarians, vegans, and those with fish allergies or aversions to fishy aftertastes. Additionally, algae cultivation in controlled environments minimizes contamination, preserving marine resources and aquatic ecosystems. This alternative reduces dependence on wild fish stocks without harming animals and enables the production of customized DHA and EPA profiles. In mice with colitis, supplementation of algal oil rich in DHA and EPA significantly inhibited the elevation of malondialdehyde, a lipid peroxidation product, and inflammatory mediator levels in the colon [15]. Results from a randomized, crossover study have suggested that DHA is more effective than EPA in modulating specific markers of inflammation in humans [16]. These findings imply that algal oil (AO) with a high content of DHA could be a valuable antioxidant and anti-inflammatory agent for treating AP [17].

To date, there has been no investigation into the impact and underlying mechanisms of AO on AP. Our study aimed to address this gap by employing a sodium taurocholate (STC)-induced injury model in pancreatic acinar cells in vitro. The objective was to explore the potent protective effects and molecular mechanisms of AO in preventing the development of AP. In this study, rat pancreatic acinar AR42J cells were subjected to AO treatment before the induction of AP. Subsequently, we assessed intracellular Ca^2+^ concentration, mitochondrial function, oxidative stress levels, and inflammatory mediators to gain insights into the preventive effects of AO on the development of AP.

## 2. Materials and Methods

### 2.1. STC-Induced AP Cell Model

Rat pancreatic acinar AR42J cells were purchased from Bioresource Collection and Research Center (BCRC, #60160, passage number 31) in Taiwan and cultured in Ham’s F-12 medium (F-12K, GIBCOTM, Thermo Fisher Scientific Inc., Waltham, MA, USA) with 2 mM L-glutamine, 20% fetal bovine serum (FBS), 100 units/L penicillin, and 100 μg/L streptomycin in a humidified atmosphere of 5% CO_2_ at 37 °C. The passage number of AR42J cells used in all experiments ranged from 40 to 48.

According to the study by Xiang et al. [18], the optimal concentration and duration of STC treatment for establishing an AP cell model using AR42J cells can achieve 50% cell viability with increased ROS production compared to the control group (no STC treatment). AR42J cells were seeded for 48 h before STC treatments at concentrations of 0, 0.5, 5, 8, 9, 10, 11, and 12 mM for 1 or 2 h. Cell viability was assessed using a flow cytometer (Becton, Dickinson and Company, BD Accuri^®^ C6, Franklin Lakes, NJ, USA), and the results are expressed as percentages relative to the control group.

To measure intracellular ROS production in AR42J cells subjected to different doses of STC for 1 or 2 h, we utilized the cell-permeant, non-fluorescent molecule 2′, 7′-dichlorodihydrofluorescein diacetate (H_2_DCFDA, Merck, Darmstadt, Germany), following the method outlined in Xu et al.’s study [19]. ROS production is expressed as the relative fold change compared to the control group.

### 2.2. AO Components, Antioxidant Activity, and Preparation

AO derived from Schizochytrium spp. was procured from DSM Nutritional Products Ltd. (Life’s DHA™ S40-O500, Kaiseraugst, Switzerland). Each gram of AO contained 204 mg of saturated fatty acids, 107 mg of monounsaturated fatty acids, and 688 mg of PUFAs, including 453 mg of DHA, 192 mg of DPA, and 15 mg of EPA. The detailed fatty acid profile of AO is provided in Table 1. Additional constituents include 89 mg of cholesterol, 2.3 mg of tocopherols, 1 mg of ash, 16 mg of calcium, and negligible amounts of other materials.

The antioxidant activity of AO was assessed using a 2,2-diphenyl-1-picryl-hydrazyl-hydrate (DPPH) free-radical scavenging assay, following the method described by Dok-Go et al. [20]. The amounts of DHA in AO treatment were 10, 20, 50, 100, 200, and 400 μM, respectively. The absorbance of DPPH was used as the control (100%). Alpha-tocopherol (20 μM in 95% ethanol) and a 95% ethanol solution were used as positive and negative controls, respectively.

In sterile conditions, AO was dissolved in an F-12K medium to achieve a stock containing 1.5 mM of DHA, 1% fatty acid-free bovine serum albumin (BSA), and 0.1% dimethyl sulfoxide (DMSO) for further experiments [21].

To evaluate the effects of AO on cell viability, AR42J cells were pretreated with medium containing 0, 10, 20, 50, 100, 200, 400, 800, and 1000 μM of DHA for 2 h, followed by serum-free medium containing 9 mM STC for 1 h. Cells treated with normal medium containing 0 or 0.1% DMSO for 2 h, followed by serum-free medium without STC for 1 h, were included as the CON and DMSO groups, respectively. The results of cell viability are expressed as percentages relative to the CON group.

### 2.3. Experimental Design

To assess the protective effects of AO on STC-induced cell damage, AR42J cells were divided into six groups. Cells in the control group (CON) and the DMSO group were cultured with normal medium and medium containing 0.1% DMSO, respectively, for 2 h, followed by serum-free medium for 1 h. In the STC group, cells were cultured with medium containing 0.1% DMSO for 2 h, followed by serum-free medium containing 9 mM STC for 1 h without AO treatment. AR42J cells in the AOL + STC, AOM + STC, and AOH + STC groups were pretreated with AO, containing 50, 100, and 150 μM of DHA, respectively, for 2 h in a medium containing 0.1% DMSO, and then subjected to STC treatment as in the STC group.

For cell lysate and medium samples, cells were cultured for 48 h and treated with AO for 2 h, followed by a 1 h exposure to a 9 mM STC treatment. Medium samples were collected and stored at −80 °C for subsequent analysis. Subsequently, cells were treated with 200 μL of cell lysis buffer (0.01 M HEPES, 1.5 mM MgCl_2_, 0.01 M KCl, 50 mM NaF, 1 mM Na_3_VO_4_, 1 mM Na_4_P2O_7_, and 0.5% NP-40) for 20 min on ice. After centrifugation, the supernatants were collected as cell lysate samples and stored at −80 °C. To standardize for cell numbers in each sample, protein concentrations of the cell lysate samples were determined using the BCA protein assay kit (Pierce, Rockford, IL, USA) with BSA as the standard.

### 2.4. Measurements

Intracellular Ca^2+^ concentration in AR42J cells was assessed using a fluorescent Fura-2 assay kit (Abcam, Eugene, OR, USA). According to the experimental design, cells underwent pretreatment with or without AO for 2 h, followed by exposure to 0 or 9 mM STC for 1 h. After the STC treatment, the medium was removed, and Fura-2 AM solution was added and incubated at 37 °C for 1 h. The fluorescence intensity was determined at 340/380 nm for excitation and 510 nm for emission using a microplate reader, following the manufacturer’s instructions. Intracellular Ca^2+^ concentration is expressed as a percentage of the CON group.

Amylase and lipase levels in the medium samples were quantified using commercially available kits obtained from Fortress Diagnostics Ltd., Antrim, UK.

Mitochondrial membrane potential (MMP) was evaluated using the potentiometric dye JC-1 from a commercial assay kit (M34152, Invitrogen^TM^, Thermo Fisher Scientific Inc., Waltham, MA, USA), following the manufacturer’s instructions.

Intracellular ROS production was assessed using H_2_DCFDA, as described previously. ROS production is presented as the relative fold change compared to the CON group. The end products of lipid peroxidation, thiobarbituric acid reactive substances (TBARS), were quantified in the cell lysate samples using the method outlined by Ohkawa et al. [22] Concentrations of TNF-α and IL-6 in the medium samples were measured using commercially available ELISA kits (R&D System, Minneapolis, MN, USA).

Cytosol and nuclear proteins were extracted using a commercial kit (NE-PER Nuclear and Cytoplasmic Extraction Kit, Thermo Fisher Scientific Inc., Waltham, MA, USA). The protein expression of total NF-κB p65 (sc-8008, Santa Cruz Biotechnology, Inc., Dallas, TX, USA), phosphorylated NF-κB p65 (p-NF-κB p65, sc-136548), and inflammasome protein nucleotide-binding domain and leucine-rich repeat pyrin domain-containing protein 3 (NLRP3, NBP2-12446, Novus Biologicals, Littleton, CO, USA) was determined through Western blotting analysis. β-actin (sc-47778) and lamin B1 (sc-374015) served as the internal controls for cytosolic and nuclear proteins, respectively. The expression of the target protein was calculated as a percentage of the CON group after normalization to the levels of β-actin or lamin B1.

### 2.5. Statistical Analysis

The differences among the six treatment groups of AR42J cells were analyzed using a one-way analysis of variance (ANOVA). Data are presented as mean ± standard error of the mean (SEM). The Fisher least-significant difference (LSD) test was used as a post hoc analysis to compare group differences when a one-way ANOVA indicated a significant group effect at *p* < 0.05. Statistical analyses were conducted using SAS 9.4 (SAS Institute, Inc., Cary, NC, USA).

## 3. Results

### 3.1. Free-Radical Scavenging Activity of AO Increased in a Dose-Dependent Enhancement

**Figure 1:**
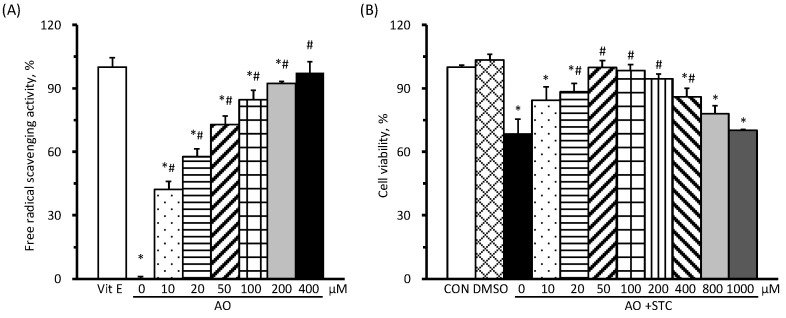
Antioxidant activity of algal oil (AO) and its effects on cell viability. (**A**) The antioxidant activity of AO, containing 10–400 μM of docosahexaenoic acid (DHA, *n* = 4 per dose), was determined using a 2,2-diphenyl-1-picryl-hydrazyl-hydrate (DPPH) radical scavenging assay. Alpha-tocopherol (Vit E, 20 μM) served as a positive control with 100% free-radical scavenging activity. (**B**) The effects of STC induction and AO pretreatment, containing 0–1000 μM of DHA (*n* = 3 per dose), on cell viability were presented as a percentage of the CON group (without STC or AO pretreatment). All values are presented as means ± SEM. * *p* < 0.05, compared with the Vit E or CON group; # *p* < 0.05, compared with STC but no AO pretreatment (one-way ANOVA with LSD).

Using 20 μM of alpha-tocopherol as a positive control for 100% DPPH free-radical scavenging activity, various amounts of AO containing DHA ranging from 0 μM (ethanol negative control) to 400 μM were tested. The results demonstrated DPPH free-radical scavenging activity of 0%, 42.3%, 57.7%, 73.1%, 84.6%, 92.3%, and 97.4% for AO containing DHA concentrations of 0, 10, 20, 50, 100, 200, and 400 μM, respectively (Figure 1A). Furthermore, the free-radical scavenging activity of AO exhibited a dose-dependent increase.

The results of different doses of AO on cell viability are shown in Figure 1B. AR42J cells in the STC group, i.e., with STC induction but no AO pretreatment, exhibited significantly decreased cell viability compared to the CON group. Conversely, those subjected to STC induction and AO pretreatment containing 50, 100, and 200 μM of DHA showed significantly increased cell viability compared to the STC group. However, cells pretreated with AO containing 200 μM of DHA exhibited more than a 5% decrease in cell viability compared to the CON group, although this difference did not reach statistical significance. Therefore, we chose to assess the effects of AO pretreatment on the microenvironment at doses containing 50, 100, and 150 μM of DHA in STC-induced AP in AR42J cells.

### 3.2. STC Decreases Cell Viability and Increases ROS Production

**Figure 2:**
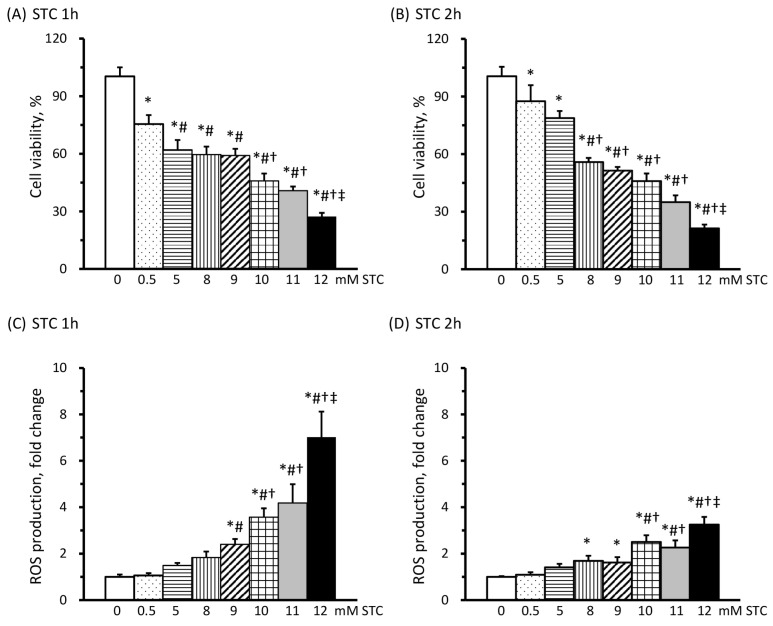
Effects of sodium taurocholate (STC) on cell viability and reactive oxygen species (ROS) production. The viability of AR42J cells exposed to 0–12 mM of STC for 1 (**A**) and 2 h (**B**) is presented as a percentage of cells without STC (100%). ROS production by AR42J cells exposed to 0–12 mM of STC for 1 (**C**) and 2 h (**D**) is depicted as fold changes relative to cells without STC (0 mM STC). All values are presented as means ± SEM; *n* = 9 per group. * *p* < 0.05 vs. 0 mM; # *p* < 0.05 vs. 0.5 mM; † *p* < 0.05 vs. 5 mM; ‡ *p* < 0.05 vs. 10 mM (one-way ANOVA with LSD).

In order to determine the optimal concentration of STC for inducing AP, the viability of AR42J cells was assessed following treatment with varying doses of STC. The results revealed a decline in cell viability as the concentrations of STC increased, both after a 1 h treatment (Figure 2A) and a 2 h treatment (Figure 2B). In comparison to cells without STC treatment, exposure to 8 and 9 mM of STC led to an approximately 50% reduction in cell viability in AR42J cells.

The production of ROS significantly increased when AR42J cells were treated with concentrations exceeding 9 mM of STC for both 1 h (Figure 2C) and 2 h (Figure 2D). Specifically, cells treated with 9–12 mM STC for 1 h displayed 2.40- to 7.02-fold increases, while those treated for 2 h showed 1.62- to 3.25-fold increases in ROS production compared to untreated cells. Additionally, ROS production was notably higher in cells treated with STC for 1 h compared to those treated for 2 h. Consequently, the decision was made to incubate AR42J cells with 9 mM STC for 1 h to induce AP, achieving approximately 50% of cell viability with a more than 2-fold increase in ROS production.

### 3.3. AO Pretreatment Enhances Cell Viability, Reduces Intracellular Calcium Ion Concentration, Improves Mitochondrial Function, and Diminishes Amylase and Lipase Secretion

**Figure 3:**
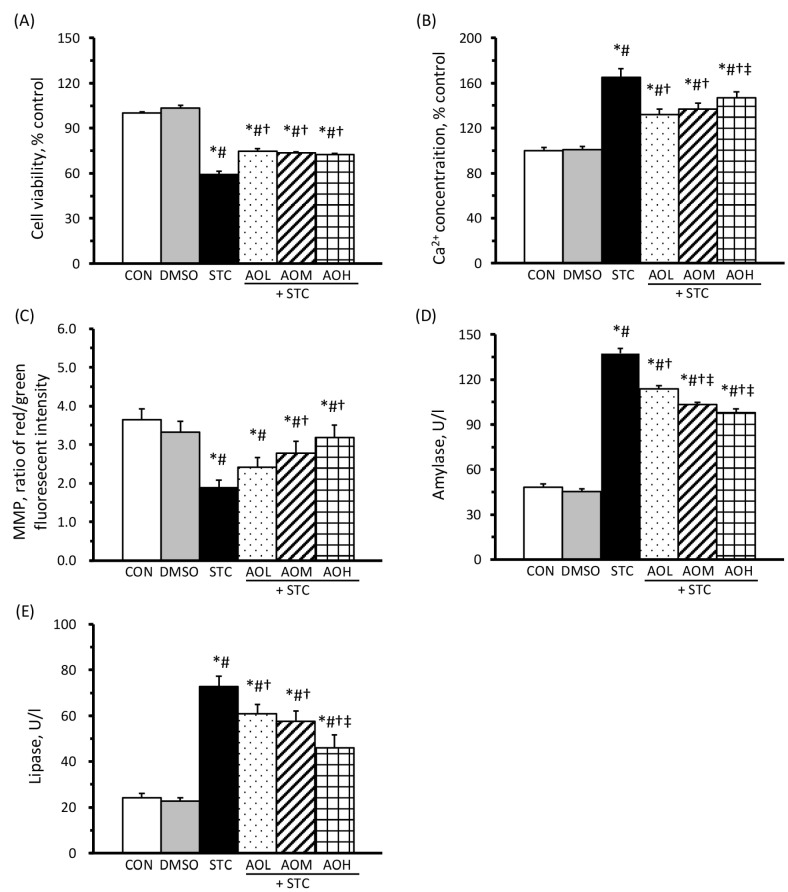
Effects of AO on cell viability (**A**), intracellular Ca^2+^ concentration (**B**), MMP (**C**), amylase (**D**), and lipase (**E**) in STC-treated cells. AR42J cells were pretreated with 0, 50, 100, or 150 μM of AO, i.e., the STC, AOL + STC, AOM + STC, or AOH + STC groups, respectively, for 2 h, followed by 9 mM STC for 1 h. The CON and DMSO groups were cultured with a normal medium and a medium containing 0.1% DMSO, respectively, without STC. Values of cell viability and intracellular Ca^2+^ concentration are presented as a percentage of the CON group (100%). MMP is presented as fold changes in the CON group. All values are presented as means ± SEM; *n* = 12 per group. * *p* < 0.05, as compared with the CON group; # *p* < 0.05, as compared with the DMSO group; † *p* < 0.05, as compared with the STC group; ‡ *p* < 0.05, as compared with the AOL group (one-way analysis of variance with least significant difference).

The results of cell viability and intracellular Ca^2+^ concentration in AR42J cells are shown in Figure 3A,B, respectively. No significant differences were observed in these parameters between the CON and DMSO groups. However, the STC group exhibited a significant decrease in cell viability and an elevation in intracellular Ca^2+^ concentration compared to the CON and DMSO groups. Furthermore, the AOL + STC, AOM + STC, and AOH + STC groups, subjected to 9 mM STC and pretreated with AO containing 50, 100, and 150 μM of DHA, respectively, showed a significant improvement in cell viability and a reduction in intracellular Ca^2+^ concentration compared to the STC group. These findings suggest that AO pretreatment effectively, or at least partially, prevented STC-induced cell death and Ca^2+^ overload in AR42J cells.

Figure 3C displays the results of MMP, a marker of mitochondrial function, in AR42J cells. No significant disparity in MMP was observed between the CON and DMSO groups. The STC group exhibited MMP levels at approximately 50% of the CON group. However, the AOM + STC and AOH + STC groups, excluding the AOL + STC group, showed a significant enhancement in MMP compared to the STC group. These results indicate that AO pretreatment significantly prevented STC-induced mitochondrial dysfunction in AR42J cells in a dose-dependent manner.

Figure 3D,E illustrate the results of amylase and lipase levels, clinical biomarkers of pancreatitis, in the culture medium. The STC group showed an approximately 3.0-fold increase in both amylase and lipase levels compared to the CON and DMSO groups. Conversely, the AOL + STC, AOM + STC, and AOH + STC groups demonstrated significantly reduced amylase and lipase levels compared to the STC group. Moreover, AO pretreatment significantly prevented the STC-induced elevation in amylase and lipase levels in a dose-dependent manner.

### 3.4. AO Pretreatment Alleviates the STC-Induced Increase in ROS, TBARS, and Pro-Inflammatory Cytokines

**Figure 4:**
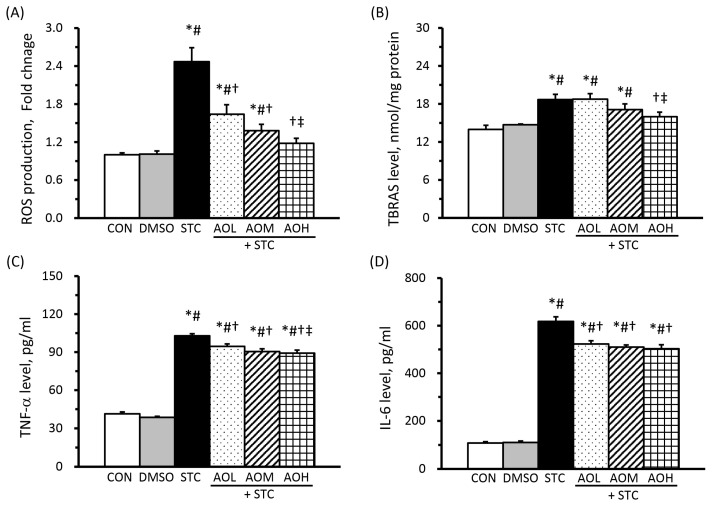
Effects of algal oil on reactive oxygen species (ROS) production (**A**) and levels of thiobarbituric acid reactive substances (TBARS, (**B**)), tumor necrosis factor (TNF)-α (**C**), and interleukin (IL)-6 (**D**) in sodium taurocholate (STC)-treated cells. AOL + STC, AOM + STC, and AOH + STC groups were pretreated with AO, containing 50, 100, and 150 μM of DHA, respectively. All values are means ± SEM; *n* = 8 to 12 per group. * *p* < 0.05 vs. the CON group; # *p* < 0.05 vs. the DMSO group; † *p* < 0.05 vs. the STC group; ‡ *p* < 0.05 vs. the AOL + STC group (one-way ANOVA with LSD).

In Figure 4A, the STC group exhibits a roughly 2.5-fold increase in ROS production compared to the CON and DMSO groups. Conversely, the AOL + STC, AOM + STC, and AOH + STC groups show significantly decreased ROS production compared to the STC group, measuring at 1.64-, 1.38-, and 1.18-fold increments over the CON group, respectively. The observed dose-dependent decrease in ROS production in AO-pretreated groups confirms the antioxidant efficacy of AO.

The levels of TBARS, indicative of lipid peroxidation end products, in the culture medium are shown in Figure 4B. The STC group exhibited significantly elevated TBARS levels compared to the CON and DMSO groups. Notably, the AOH-STC group, as opposed to the AOL + STC and AOM + STC groups, showed significantly reduced TBARS levels compared to the STC group. The observed decline in TBARS production in AO-pretreated groups follows a dose-dependent pattern.

Figure 4C,D illustrate the production of TNF-α and IL-6 in the culture medium. No significant differences were observed in TNF-α and IL-6 levels between the CON and DMSO groups. However, the STC group demonstrated approximately 2.5- and 5.7-fold increases in TNF-α and IL-6, respectively, compared to the CON group. In contrast, AO pretreatment mitigated inflammatory responses, as evidenced by significantly lower TNF-α and IL-6 levels in the AO-treated groups compared to the STC group.

### 3.5. AO Pretreatment Mitigates the STC-Induced Activation of the NF-κB Signaling Pathway

**Figure 5:**
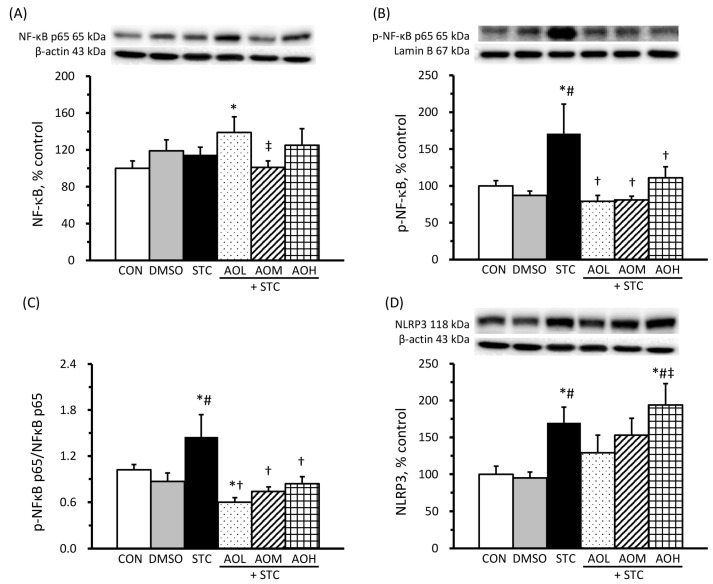
Effects of algal oil on protein levels of cytosolic NF-κB (**A**) and nucleic p-NF-κB (**B**), the ratio of nucleic p-NF-κB to cytosolic NF-κB (**C**), and NRLP-3 (**D**) in sodium taurocholate (STC)-treated cells. AOL + STC, AOM + STC, and AOH + STC groups were pretreated with AO, containing 50, 100, and 150 μM of DHA, respectively. Protein levels are presented as a percentage of the CON group (100%). The ratio of nucleic p-NF-κB to cytosolic NF-κB is presented as fold changes of the CON group. All values are means ± SEM; *n* = 8 per group. * *p* < 0.05 vs. the CON group; # *p* < 0.05 vs. the DMSO group; † *p* < 0.05 vs. the STC group; ‡ *p* < 0.05 vs. the AOL + STC group (one-way ANOVA with LSD).

The protein levels of cytosolic NF-κB p65, nuclear p-NF-κB p65, cytosol NLRP3, and the ratio of nuclear p-NF-κB p65 to cytosolic NF-κB p65 are shown in Figure 5. In Figure 5A, there is no significant difference in cytosolic NF-κB p65 protein levels among the CON, DMSO, and STC groups. AO pretreatment does not significantly impact cytosolic NF-κB p65 protein levels in STC-treated cells. However, the protein levels of nuclear p-NF-κB p65 (Figure 5B) and the ratio of nuclear p-NF-κB p65 to cytosolic NF-κB p65 (Figure 5C) showed a significant increase in the STC group compared with the CON and DMSO groups; these increases are significantly reduced in the AOL + STC, AOM + STC, and AOH-STC groups. These results indicate that AO pretreatment effectively prevented STC-induced activation of the NF-κB signaling pathway. In Figure 5D, the protein levels of the NLRP3 inflammasome in the STC and AOH + STC groups increased significantly compared with those in the CON and DMSO groups, while no significant change was observed in the AOL + STC and AOM + STC groups.

## 4. Discussion

Preventing the progression and recurrence of AP remains a formidable challenge in clinical settings. In our study involving STC-treated pancreatic acinar cells, AO pretreatment demonstrated a marked reduction in cell death, alleviated Ca^2+^ overload, mitigated mitochondrial dysfunction, and decreased the production of amylase and lipase. Furthermore, AO pretreatment notably prevented the heightened levels of ROS, pro-inflammatory cytokines, and lipid peroxidation products induced by STC. These beneficial changes were correlated with the suppression of NF-κB activation. Our results underscore the potential impact of AO in averting the onset and development of AP.

In the early stages of AP, intracellular Ca^2+^ overload plays an important role, initiating inflammation and triggering the secretion of digestive enzymes such as amylase and lipase in pancreatic acinar cells [23]. Previous studies have established a close association between intracellular Ca^2+^ overload, mitochondrial dysfunction, and the excessive production of ROS during AP progression [8,9,24,25]. The heightened ROS production further activates the NF-κB signaling pathway, initiating local inflammation in the pancreas by upregulating pro-inflammatory cytokines like TNF-α and IL-6 and initiating the NLRP3 inflammasome to trigger the inflammatory cascade in AP [26].

AR42J cells, derived from rat pancreatic acinar cells, have proven to be a valuable tool in various studies exploring pancreatic function, cell biology, and disease mechanisms [18,24,27]. In this study, AR42J cells treated with 9 mM of STC for 1 h showed a significant increase in intracellular Ca^2+^ concentration, approximately a 50% reduction in cell viability, a decrease in MMP (Figure 3C), and heightened ROS production compared to untreated cells. Additionally, the STC-treated cells showed a substantial increase in the production of amylase and lipase, as well as in TBARS, TNF-α, and IL-6, accompanied by the activation of the NF-κB signaling pathway. These observed changes closely parallel the characteristics documented in the context of AP across cellular, animal, and human studies [6,7,8,9].

Substances with antioxidant and anti-inflammatory properties have demonstrated positive effects in AP [14,16,17]. In studies involving STC-injured AR42J cells and rats, vitamin C proved effective in attenuating oxidative stress. It achieved this by reducing the lipid peroxidation product malondialdehyde, enhancing antioxidant enzyme activity and total antioxidant capacity, and mitigating the inflammatory response through a reduction in the production of IL-1β and IL-6 [27]. Furthermore, evidence suggests that PUFAs found in fish oils, particularly DHA and EPA, can play a crucial role in reducing oxidative stress and inflammatory responses in severe AP rats [14]. Pretreating with DHA has exhibited encouraging outcomes across diverse cell types and animal models. These effects encompass enhancements in calcium homeostasis, MMP, antioxidant enzyme functions, and a decrease in mitochondrial ROS production [28,29,30].

Studies on neonatal mice with brain hypoxia-ischemia reveal that DHA, in contrast to EPA, significantly attenuated Ca^2+^-induced mitochondrial permeabilization, reduced ROS production, and improved Ca^2+^ buffering disability [29]. Additionally, investigations by Yang et al. [31] demonstrated that DHA pretreatment at concentrations of 50 and 100 μM effectively mitigated inflammation by inhibiting the NF-κB signaling pathway and NLRP3 activation in human endothelial cells. In a caerulein-induced AP model characterized by mild, non-lethal pancreatitis without multiple organ dysfunction, DHA pretreatment led to decreased NF-κB activation, IL-6, lipid peroxide production, and inflammatory cell infiltration in the pancreas [32]. Collectively, these studies underscore the significant potential of antioxidant agents in alleviating oxidative stress and inflammatory responses in AP.

Algal oil, derived from algae, exhibits a dose-dependent enhancement in free-radical scavenging activity, as demonstrated by the DPPH assay. Despite the acknowledged antioxidant properties of AO, its impact and underlying mechanisms on AP have not been explored until now. In this study, AO pretreatment significantly reduced ROS production and TBARS levels in AR42J cells under STC insult. This decline in oxidative stress is associated with a reduction in intracellular Ca^2+^ concentration, an increase in MMP, and a decrease in the inflammatory response, as indicated by lower production of TNF-α and IL-6 levels.

In Figure 3 and Figure 4, our findings indicated a less pronounced reduction in pancreatic enzyme (amylase and lipase) and inflammatory cytokine (TNF-α and IL-6) levels compared to ROS and TBARS reduction, especially among groups pretreated with 100 µM to 150 µM of AO. This discrepancy may stem from the longer duration required for protein synthesis compared to free radical generation. Thus, the 2 h AO pretreatment followed by 1 h exposure to 9 mM STC may not allow sufficient time to detect significant differences in protein levels among AO-treated groups, especially under conditions of similar cell viability. It is worth noting that the levels of TNF-α, but not IL-6, exhibited a dose-dependent decrease. This could be attributed to the earlier production of TNF-α compared to IL-6 during inflammation. Additionally, significant differences were observed between groups pretreated with 50 µM and 150 µM of AO rather than 100 µM and 150 µM. This variance could be attributed to the disparity between a 3-fold increase and a 1.5-fold increase in DHA.

Examining the mechanism of AO pretreatment in mitigating inflammatory responses, prior research suggests that DHA, a component of AO, may alleviate inflammation by inhibiting the NF-κB signaling pathway and the NLRP3 inflammasome [31,33]. NF-κB activation can directly induce pro-inflammatory cytokine expression and contribute to NLRP3 inflammasome activation [34]. Consistent with previous observations, we noted that AO pretreatment resulted in the inactivation of the NF-κB signaling pathway. This was evidenced by a substantial reduction in nuclear p-NF-κB p65 protein levels and the ratio of nuclear p-NF-κB p65 to cytosolic NF-κB p65 in STC-treated AR42J cells. In contrast to earlier investigations involving different cell types, this study observed significant NLRP3 inflammasome activation in AR42J cells treated with STC. However, AO pretreatment did not exhibit a significant impact on its activation. This may be due to the cellular damage caused by STC in AR42J cells, not only inducing an NF-κB signaling pathway-medicated inflammatory response but possibly also triggering the NLRP3 inflammasome through other pathways. The activation signal for the NLRP3 inflammasome involves exposure to microbial components or endogenous cytokines. This signal primes the inflammasome by regulating the NF-κB pathway, ultimately leading to the activation of the NLRP3 inflammasome. However, the activation of the NLRP3 inflammasome could also be triggered by various molecular or cellular events, including ionic flux, mitochondrial dysfunction, ROS production, and lysosomal damage [35]. Collectively, the reduced NF-κB signaling pathway activation, coupled with decreased TNF-α and IL-6 production, suggests that AO pretreatment may primarily prevent STC-induced inflammation through modulation of the NF-κB signaling pathway in pancreatic acinar cells.

Our study possesses several notable strengths. Firstly, it represents the initial investigations into the impact and underlying mechanisms of AO, the algae-derived source of DHA, on STC-induced AP. Secondly, AO stands out as an advantageous natural resource sourced from algae, which can be industrially fermented, providing a sustainable and economically viable source of DHA. Thirdly, AO distinguishes itself by lacking cholesterol, presenting no fishy taste, and being suitable for diverse dietary preferences across various cultures. Furthermore, the concentrations of DHA in AO doses used in this study are 50, 100, and 150 μM, falling within the reported range of human plasma concentrations in young, healthy Canadian adults (7.2 to 237.5 μM) [36]. Nevertheless, there are some limitations to this study. We did not employ pure DHA as a positive control, leaving the possibility that minor components, such as specific phytochemicals and α-tocopherol, may contribute to its protective effects. In addition, we did not use Ca^2+^ chelators/additives or antioxidants to confirm whether AO’s protective effects on AP are specifically attributed to mitigated intracellular Ca^2+^ overload, oxidative stress, or a combination of both factors. Finally, the use of our cell model may not precisely reflect the complicated conditions seen in patients with AP. Therefore, further exploration of the beneficial effects of AO in the management of AP through animal and human studies is warranted.

## 5. Conclusions

Using STC to stimulate bile acid obstruction-induced AP, our findings demonstrate that AO pretreatment holds significant promise in mitigating key aspects of AP damage, including Ca^2+^ overload, mitochondrial dysfunction, oxidative stress, pancreatic enzyme production, and inflammation in pancreatic acinar cells. Furthermore, the anti-inflammatory properties of AO are mediated through the inhibition of NF-κB activation. These findings underscore the potential protective value of AO in preventing AP. Additional in vivo and clinical studies are crucial to further exploring AO’s efficacy in different pancreatitis etiologies, assessing its safety, and determining the optimal dosage. These efforts are essential for translating the promising preclinical results of AO obtained in this study into practical applications.

## Figures and Tables

**Table 1:** Fatty acid profile of algal oil used in the present study.

Type of Fatty Acids	g/100 g	Type of Fatty Acids	g/100 g
Total fat	**99.9**	Polyunsaturated fatty acids	
Saturated fatty acids	20.4	Omega 6 fatty acids	**21.8**
Monounsaturated fatty acids	10.7	18:2 n6 Linoleic acid	0.4
Polyunsaturated fatty acids	68.6	18:3 n6 γ-Linolenic acid	0.3
Trans fatty acids	0.2	20:2 n6 Eicosadienoic acid	0
		20:3 n6 hommo-γ-Linolenic acid	0.5
Saturated fatty acid		20:4 n6 Arachidonic acid	1.4
12:0 Lauric acid	0.1	22:5 n6 Docosapentaenoic acid (DPA)	19.2
14:0 Myristic acid	4.6		
16:0 Palmitic acid	14.6	Omega 3 Fatty Acids	**46.8**
18:0 Stearic acid	0.6	18:3 n3 α-Linolenic acid	0
		20:5 n3 Eicosapentaenoic acid (EPA)	1.5
Monounsaturated fatty acid		22:5 n3 Docosapentaenoic acid (DPA)	0
16:1 Palmitoleic acid	0.1	22:6 n3 Docosahexaenoic acid (DHA)	45.3
17:1 Heptadecenoic acid	0.2		
18:1 n9 Oleic acid	9.4		
18:1 n7 Vaccenic acid	0.2		

Information was sourced from DSM Nutritional Products Ltd. (Life’s DHA™ S40-O500, Kaiseraugst, Switzerland).

## Data Availability

The data presented in this study are available on request from the corresponding author.
